# Hierarchical VOOH hollow spheres for symmetrical and asymmetrical supercapacitor devices

**DOI:** 10.1098/rsos.171768

**Published:** 2018-01-31

**Authors:** Xuyang Jing, Cong Wang, Wenjing Feng, Na Xing, Hanmei Jiang, Xiangyu Lu, Yifu Zhang, Changgong Meng

**Affiliations:** 1 College of Chemistry and Chemical Engineering, Liaoning Normal University, Dalian 116029, People's Republic of China; 2 School of Chemistry, Dalian University of Technology, Dalian 116024, People's Republic of China

**Keywords:** VOOH, hierarchical structures, hollow spheres, electrochemical properties, supercapacitor, device

## Abstract

Hierarchical VOOH hollow spheres with low crystallinity composed of nanoparticles were prepared by a facile and template-free method, which involved a precipitation of precursor microspheres in aqueous solution at room temperature and subsequent hydrothermal reaction. Quasi-solid-state symmetric and asymmetric supercapacitor (SSC and ASC) devices were fabricated using hierarchical VOOH hollow spheres as the electrodes, and the electrochemical properties of the VOOH//VOOH SSC device and the VOOH//AC ASC device were studied by cyclic voltammetry (CV), galvanostatic charge–discharge (GCD) and electrochemical impedance spectroscopy (EIS). Results demonstrated that the electrochemical performance of the VOOH//AC ASC device was better than that of the VOOH//VOOH SSC device. After 3000 cycles, the specific capacitance of the VOOH//AC ASC device retains 83% of the initial capacitance, while the VOOH//VOOH SSC device retains only 7.7%. Findings in this work proved that hierarchical VOOH hollow spheres could be a promising candidate as an ideal electrode material for supercapacitor devices.

## 1. Introduction

Energy storage devices including Li-ion batteries (LIBs), Na-ion batteries (NIBs), supercapacitors and fuel cells have played an important role as energy resources applied to various electronic devices such as portable and wearable electronics, hybrid electric vehicles, bio-implantable devices and LED devices [1–10]. Among them, supercapacitors (SCs), also known as electrochemical capacitors or ultracapacitors, have drawn tremendous interest due to their superior properties of excellent power output, fast ion delivery rate, long cycle life, lightweight, ease of handing, etc. which can complement LIBs and NIBs [11–16]. SCs store the energy through two operating mechanisms. Electrochemical double layer capacitance (EDLC) physically stores charges resulting from the electrical double layer surrounding the surface of the electrode. Pseudo-capacitance (PC) chemically stores charges originating from the redox reaction of the electrode material with the electrolyte [13,17]. However, the low energy density and the difficulty involved in integrating these SCs into a circuit for a self-operating system are challenges to their actual application [4,11,18]. As is well known, the energy density (*E*) of the SC device is governed by the specific capacitance (*C*) and output voltage (*V*) according to the relationship *E* = 1/2*CV*^2^. Therefore, the efficient strategy to improve the energy density of an SC device is to increase the specific capacitance or output voltage of the designed SC device.

In the past decades, vanadium oxides and their related materials applied to energy storage have been extensively studied for electrode materials due to their low cost, multiple valences, novel chemical and physical properties, and high specific capacity [19–25]. Very recently, vanadyl hydroxide (VOOH) has received increasing attention with regard to its synthesis and properties. Xie's group prepared various VOOH structures such as hollow dandelions [26], single-shelled/double-shelled hollow nanospheres [27], quadrangular nanorods [28] and hollow nanourchins [29]. These reports were focused on the synthesis and shape control as well as the application to lithium-ion batteries [26], electrical switch [28] and the conversion to VO_2_ [29,30]. Thereafter, Shao *et al*. [31] applied VOOH hollow microspheres to NIBs and found that VOOH exhibited outstanding rate behaviour and long life. Zhu & Ruan [32] synthesized groove-like VOOH nanostructures by an in-suit Kirkendall effect and oriented attachment process and applied these structures to aqueous LIBs. Wang *et al*. [33] reported the applications of lepidocrocite VOOH in electrocatalytic water splitting, and the results demonstrated VOOH hollow nanospheres to be an efficient alternative to water splitting. At present, our group focus on the application of VOOH to an SC's electrode for energy storage. In our previous reports, we synthesized hierarchical porous VOOH hollow spheres and studied their electrochemical properties as an SC's electrode in aqueous and organic electrolytes [34,35]. Results demonstrated that hierarchical VOOH hollow spheres featured capacitive behaviour based on PC, indicating VOOH is an ideal material for an SC's electrodes. However, to the best of our knowledge, the applications of the as-obtained hierarchical VOOH hollow spheres to symmetric SC (SSC) or asymmetric SC (ASC) devices have not been studied. Herein, we put emphasis on the electrochemical performance of hierarchical VOOH hollow spheres applied to SSC or ASC devices.

In this contribution, we investigated the electrochemical properties of hierarchical VOOH hollow spheres as the electrode material applied to SSC or ASC devices. Findings demonstrated that the electrochemical performance of the VOOH//AC ASC device was better than that of the VOOH//VOOH SSC device.

## 2. Experimental section

### 2.1. Synthesis and characterization

**Figure 1.**
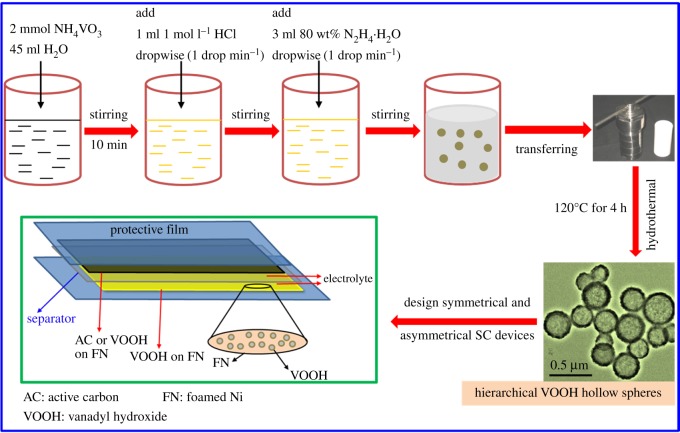
A schematic illustration of the synthesis of hierarchical VOOH hollow spheres for designedly fabricating symmetrical and asymmetrical SC devices.

All the chemicals of analytical grade were purchased from Sinopharm Chemical Reagent Co., Ltd and used without any further purification. Figure 1 depicts the synthesis of hierarchical VOOH hollow spheres for designedly fabricating symmetrical and asymmetrical SC devices. The synthesis of hierarchical VOOH hollow spheres was based on our previous reports [34,35]. In detail, 0.234 g of NH_4_VO_3_ and 45 ml of distilled water were added to a 100 ml beaker with strong stirring. Then, 1 ml of 1.0 mol l^−1^ HCl solution was dropped into the above solution. After the solution became a transparent yellow solution, 3 ml of N_2_H_4_ · H_2_O was added to the above solution and stirred for 30 min at room temperature. In the process, the transparent solution became a suspension and the colour changed from yellow to grey. The grey precipitations were V(OH)_2_NH_2_ solid spheres [27,35]. Finally, the above solution was transferred into a Teflon-lined stainless steel autoclave, sealed and maintained at 120°C for 4 h. After the reaction, the products were filtered off, washed with distilled water and ethanol several times to remove any possible residue, and dried at 75°C for 12 h in vacuum. Phase, composition and morphology of the as-obtained hierarchical VOOH hollow spheres have been reported and seen in our previous reports [34,35]. The active carbon we used was ordinary commercial active carbon (YP-50F) purchased from Kurary. Its BET specific surface area is 1666 m^2^ g^−1^.

### 2.2. Fabrication and electrochemical characterization of symmetric and asymmetric supercapacitor devices

The working electrodes were prepared using a mixture of 80 wt% of the as-synthesized hierarchical VOOH hollow spheres, 10 wt% of polyvinylidene difluoride (PVDF) and 10 wt% of carbon black, and *N*-methyl-2-pyrrolidone (NMP) was used as a solvent. The mixed slurries were coated onto formed Ni (FN) and heated at 80°C overnight to remove the organic solvent. Then these foils were pressed onto Ni-grids at a pressure of 10 MPa to make electrodes. The mass loading of hierarchical VOOH hollow spheres was about 4 mg cm^−2^. To fabricate the negative electrode, we used commercial activated carbon (AC) replacing the as-synthesized hierarchical VOOH hollow spheres with the same process. The electrolyte used was an organic electrolyte (lithium perchlorate in propylene carbonate, 1 mol l^−1^).

The fabrication of the device is illustrated in figure 1. The quasi-solid-state asymmetric SC (ASC) was assembled using hierarchical VOOH hollow spheres as the positive electrode, AC as the negative electrode and a separator (NKK-PF30AC) sandwiched in between. The device is denoted as VOOH//AC ASC device. After each electrode dipped into the electrolyte, the entire device was sealed in a plastic sheet to avoid the evaporation of the electrolyte. To optimize the performance of the device, the mass ratio of the positive to negative electrode is balanced on the basis of the equal charge between the cathode and the anode by using the equation below:

2.1 $$\frac{m_{+}}{m_{-}}=\frac{\left(C_{-}\cdot \Delta V_{-}\right)}{\left(C_{+}\cdot \Delta V_{+}\right)}.$$

The quasi-solid-state symmetric SC (SSC) was assembled using the same process with the VOOH hollow spheres electrode replacing the AC electrode. The device is denoted as the VOOH//VOOH SSC device. Cyclic voltammetry (CV), galvanostatic charge–discharge (GCD) and electrochemical impedance spectroscopy (EIS) were used to investigate the electrochemical performance of the assembled devices. The areal capacitance was calculated on the basis of GCD curves according to the following equation:

2.2 $$C=\frac{I\cdot \Delta t}{s\cdot \Delta V},$$

where *C* (mF cm^−2^) represents the specific capacitance; *I* (A) denotes the discharge current; Δ*t* (s) refers to the discharge time; *s* (cm^2^) corresponds to the working area of the electrode; and Δ*V* (V) represents the potential windows during the discharge process. The areal energy density *E* (W h m^−2^) and power density *P* (W m^−2^) of the devices can be calculated according to equations (2.3) and (2.4). Please note the conversion of the unit during the calculation:

2.3 $$E=\frac{1}{2}C\cdot {\left(\Delta V\right)}^{2}$$

and

2.4 $$P=\frac{E}{\Delta t}.$$

## 3. Results and discussion

Figure 1 illustrates the successful synthesis of hierarchical VOOH hollow spheres by a facile and template-free hydrothermal route. The as-obtained VOOH was the lepidocrocite phase with low crystallinity according to our previous reports [34,35]. Hollow spheres with hollow cores and VOOH shells are clearly observed in FE-SEM and TEM images [34,35]. VOOH hollow spheres have a rough surface comprising lots of nanoparticles. These characteristics indicate that VOOH hollow spheres might possess high specific surface area and hierarchical structure, which are proved by nitrogen adsorption–desorption isotherms [35]. The BET surface area of VOOH hollow spheres reaches 32 m^2^ g^−1^. VOOH hollow spheres possess macropores from the hollow interior and mesopores from the VOOH shell, with the most probable distribution pore size of VOOH hollow spheres measuring 3.6 nm. The diameter of hierarchical VOOH hollow spheres is in the range of 300–500 nm, and the shell thickness is 20 nm on average. Therefore, hierarchical VOOH hollow spheres with low crystallinity of the VOOH shells, consisting of nanoparticles and hollow cores, are prepared. The formation of hierarchical VOOH hollow spheres undergoes an inside-out Ostwald ripening [27]. Such structures indicate that hierarchical VOOH hollow spheres probably exhibit excellent electrochemical performance that can be applied to SSC and ASC devices [36].

**Figure 2.**
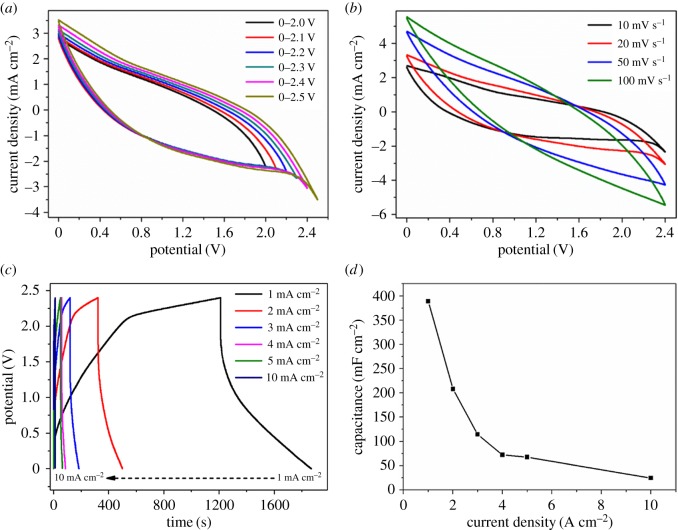
Electrochemical performance of the VOOH//VOOH SSC device: (*a*) CV curves in different potential windows at 10 mV s^−1^; (*b*) CV curves at different scan rates; (*c*) GCD curves at different current densities; (*d*) the corresponding areal capacitance from GCD curves.

To evaluate the practical application of hierarchical VOOH hollow spheres, quasi-solid-state SSC and ASC devices (figure 1) were assembled. Figure 2 represents the electrochemical performance of a VOOH//VOOH SSC device assembled using the as-obtained hierarchical VOOH hollow spheres. As shown in figure 2*a*, a series of CV curves are tested in different potential windows to determine the optimal operating potential window of the VOOH//VOOH SSC device. The optimal potential window of the VOOH//VOOH SSC device is determined to be 2.4 V due to the largest specific capacitance at this window. Figure 2*b* shows CV curves of the VOOH//VOOH SSC device at different scan rates in the potential window of 2.4 V. These CV curves exhibit a quasi-rectangular shape and no obvious deformation is observed at high scan rates, indicating the ideal capacitive behaviour of the VOOH//VOOH SSC device. GCD curves of the VOOH//VOOH SSC device at different current densities are shown in figure 2*c* and the corresponding areal capacitance is calculated from GCD curves as a function of current density, as depicted in figure 2*d*. According to equation (2.2), the full VOOH//VOOH SSC device delivers areal capacitances of 389, 208, 114, 72, 68 and 25 mF cm^−2^ at current densities of 1, 2, 3, 4, 5 and 10 mA cm^−2^, respectively. The VOOH//VOOH SSC device displays rate capability, with 6.4% of the areal capacitance (the value at 10 mA cm^−2^) retained compared with the value at 1 mA cm^−2^.

**Figure 3.**
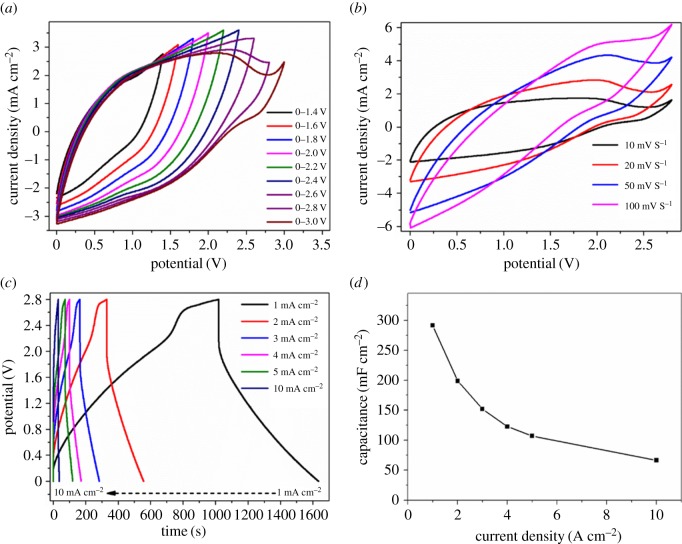
Electrochemical performance of the VOOH//AC ASC device: (*a*) CV curves in different potential windows at 10 mV s^−1^; (*b*) CV curves at different scan rates; (*c*) GCD curves at different current densities; (*d*) the corresponding areal capacitance from GCD curves.

Figure 3 shows the electrochemical performance of the designed VOOH//AC ASC device assembled using the as-obtained hierarchical VOOH hollow spheres and AC as the cathode and the anode, respectively. AC shows the typical double layer capacitance and it is widely used as the electrode material applied to SCs [37]. The potential window of AC is usually at −1–0 V as an SC electrode [9], and the potential window of VOOH at −0.1–0.9 V [35]. Those two materials exhibit entirely different potential windows, which can maximize the operating cell voltage of the VOOH//AC ASC device. Figure 3*a* shows a series of CV curves in different potential windows to determine the optimal operating potential window of the VOOH//AC ASC device. The optimal potential window of the VOOH//AC ASC device is determined to be 2.8 V owing to the largest specific capacitance at this window, which is larger than that of the VOOH//VOOH SSC device. Figure 3*b* represents CV curves of the VOOH//AC ASC device at different scan rates in the potential window of 2.8 V. These CV curves display a quasi-rectangular shape and no obvious deformation is observed at high scan rates, indicating the ideal capacitive behaviour of the VOOH//AC ASC device in agreement with the capacitive behaviour of hierarchical VOOH hollow spheres and AC. GCD curves of the VOOH//AC ASC device at different current densities are depicted in figure 3*c,* and the corresponding areal capacitance calculated from GCD curves as a function of current density is shown in figure 3*d*. According to equation (2.2), the full VOOH//AC ASC device exhibits areal capacitances of 292, 199, 152, 123, 108 and 67 mF cm^−2^ at current densities of 1, 2, 3, 4, 5 and 10 mA cm^−2^, respectively. The VOOH//AC ASC device shows higher rate capability than that of the VOOH//VOOH SSC device.

Table 1 summarizes the electrochemical performance of the VOOH//VOOH SSC device and the VOOH//AC ASC device in this study and the previous values reported in the references. The specific capacitances of the VOOH//VOOH SSC device and the VOOH//AC ASC device in this work have higher capacitance than the values in the literature. The specific capacitance of the VOOH//VOOH SSC device is higher than that of the VOOH//AC ASC device in the lower current densities; however, in the higher current densities, the VOOH//AC ASC device exhibits better electrochemical performance than the VOOH//VOOH SSC device, as shown in figure 2*d* and figure 3*d*.

**Table 1.** Comparison of the electrochemical performance of SC devices. M, mol l^−1^; PVA, polyvinyl alcohol; PC, propylene carbonate.

types of materials	mass loading of the electrode (mg)	electrolyte	potential (V)	capacitance (mF cm^−2^) (test condition)	E (W h m^−2^)	*P* (W m^−2^)	reference
V_2_O_5_ SSC (device)	—	1 M LiClO_4_/PVA	0–1.8	380, 1 mV s^−1^	—	—	[38]
V_2_O_5_ H_2_O/graphene SSC	2.5	LiCl/PVA	−0.8–0.8	11.72, 0.25 A m^−2^	1.14 × 10^−2^	0.1	[39]
VO_2_ NF@3DG SSC	8.22	0.5 M K_2_SO_4_	−0.6–0.6	70.8, 0.5 mA cm^−2^	0.280	6	[40]
V_2_O_5_ SSC (device)	4.02∼4.13	LiCl/PVA	1.0	144.1, 5 mA cm^−2^	—	—	[41]
VO_2_(A)@C//AC (device)	4	1 M Na_2_SO_4_	0–1.5	228, 0.5 mA cm^−2^	0.714	3.75	[10]
V_2_O_3_@C//AC (device)	—	5 M LiCl/PVA	0–0.8	297, 0.5 mA cm^−2^	0.334	2.25	[42]
VOOH SSC (device)	4	1 M LiClO_4_/PC	0–2.4	389, 1 mA cm^−2^	3.11	6	this work
VOOH//AC ASC (device)	4	1 M LiClO_4_/PC	0–2.8	292, 1 mA cm^−2^	3.17	7	this work

**Figure 4.**
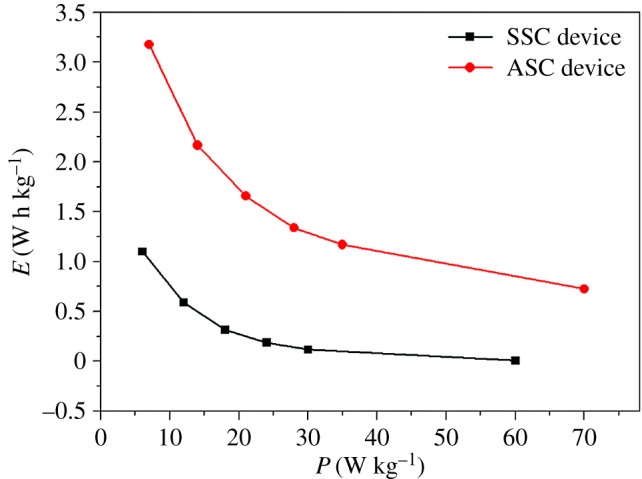
Ragone plots of the VOOH//VOOH SSC device and the VOOH//AC ASC device.

**Figure 5.**
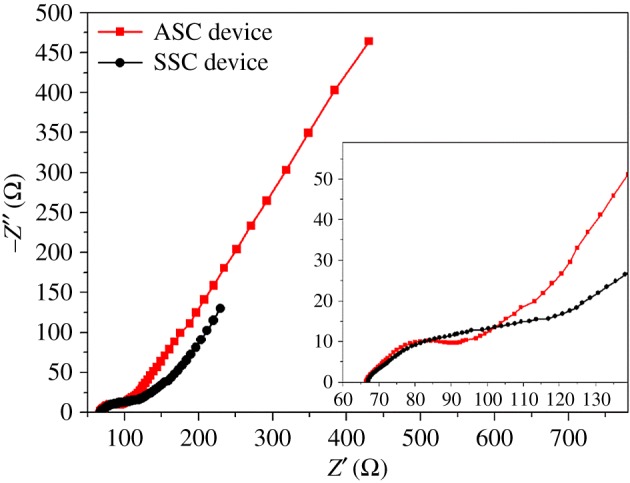
Nyquist plots of the VOOH//VOOH SSC device and the VOOH//AC ASC device in the frequency range from 100 kHz to 0.01 Hz.

Figure 4 shows the Ragone plots of the VOOH//VOOH SSC device and the VOOH//AC ASC device. For the VOOH//VOOH SSC device, the calculated energy density *E* of the device at the current density of 1, 2, 3, 4, 5 and 10 mA cm^−2^ is 3.11, 1.67, 0.92, 0.58, 0.54 and 0.20 W h m^−2^ and the corresponding power density *P* is 6, 12, 18, 24, 30 and 60 W m^−2^, respectively. For the VOOH//AC ASC device, the calculated *E* of the device at the current density of 1, 2, 3, 4, 5 and 10 mA cm^−2^ is 3.18, 2.16, 1.66, 1.34, 1.17 and 0.72 W h m^−2^ and the corresponding *P* is 7, 14, 21, 28, 35 and 70 W m^−2^, respectively. Thus, it is clearly shown (figure 4) that the electrochemical performance of the VOOH//AC ASC device is better than that of the VOOH//VOOH SSC device, which is very consistent with the results of GCD. Table 1 lists the comparative energy densities and power densities of our values and the reported values related with vanadium-based materials and other materials. Results obviously display that the VOOH//VOOH SSC device and the VOOH//AC ASC device have excellent electrochemical properties. To disclose why the VOOH//AC ASC device exhibits excellent electrochemical behaviour, EIS was carried out over a frequency range of 100 kHz to 0.01 Hz, as depicted in figure 5. The Nyquist plots comprise a semicircle and an inclined line. The semicircle diameter (Rct) represents the charge-transfer resistance and the intercept of the semicircle (Re) denotes the electrolyte resistance. The inclined line is associated with ion diffusion kinetics [43]. In figure 5, the slope of the VOOH//AC ASC device is larger than that of the VOOH//VOOH SSC device, indicating that the VOOH//AC ASC device has enhanced electronic and ionic conductivities. The small diameter of the semicircle and the high slope in the Nyquist plot of the VOOH//AC ASC device indicate that the charge-transfer resistance of the device is small and the diffusion of ions is fast [44]. These much-improved electronic and ionic conductivities lead to better electrochemical performances.

**Figure 6.**
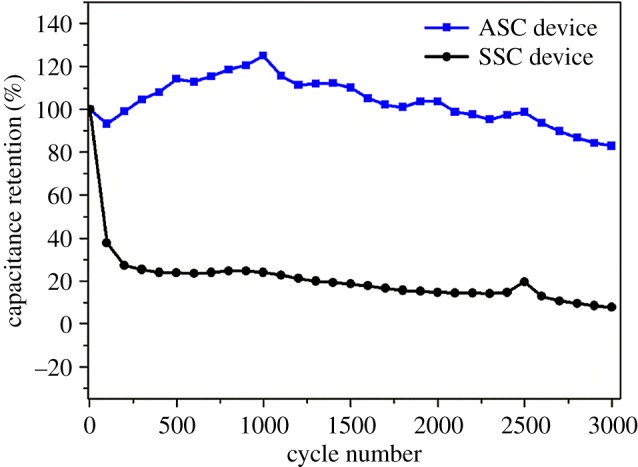
The cycling performance of the as-fabricated VOOH//VOOH SSC device and VOOH//AC ASC device at 3 mA cm^−2^ for 3000 cycles.

Figure 6 describes the cycling performance of the as-fabricated VOOH//VOOH SSC device and the VOOH//AC ASC device at 3 mA cm^−2^ for 3000 cycles. It can be obviously observed that the cycling performance of the VOOH//AC ASC device is much better than that of the VOOH//VOOH SSC device. For the VOOH//VOOH SSC device, the specific capacitance quickly fades in the first 100 cycles. It only retains 37.7% of the initial capacitance. The specific capacitance gradually decreases to 7.7% of the initial capacitance after 3000 cycles. However, for the VOOH//AC ASC device, in the first 100 cycles, the specific capacitance slowly decreases to 93.2% of the initial capacitance. Then the specific capacitance slightly increases with the increase in the number of cycles. After 1000 cycles, the capacitance retention reaches 125%, suggesting that the highest areal capacitance is achieved. Gradual improvement of the areal capacitance of the VOOH//AC ASC device indicates the gradual penetration of electrolytes of the interior of hollow spheres, demonstrating high rate capacitance of the electrodes consisting of hierarchical VOOH hollow spheres, in agreement with the results discussed in figure 2 and figure 3 [45]. The above observation corresponds with the electrochemical performance of the VOOH single electrode reported in the literature [35]. After 1000 cycles, the specific capacitance slightly decreases and it retains 83% of the initial capacitance after 3000 cycles, which is higher than that of the VOOH//VOOH SSC device. Based on the above results, the electrochemical performance of the VOOH//AC ASC device is better than that of the VOOH//VOOH SSC device. The exact reason for this difference is unknown at present; however, comparing these two devices, the reason may be due to AC, because it always shows a good performance in cycling ability [46]. However, the reason is still being studied in our further research.

## 4. Conclusion

In summary, hierarchical VOOH hollow spheres were explored to fabricate the quasi-solid-state SSC and ASC devices, and their electrochemical properties were studied by CV, GCD and EIS. Results display that the VOOH//VOOH SSC device and the VOOH//AC ASC device have excellent electrochemical properties. Furthermore, the electrochemical performance of the VOOH//AC ASC device is better than that of the VOOH//VOOH SSC device. The VOOH//AC ASC device exhibits a larger potential window, higher capacitance and rate capacitance and longer cycling performance than those of the VOOH//VOOH SSC device. The present study provides insights into exploration of new materials that can be used as electrodes for SSC and ASC applications. Future work will focus on why the VOOH//AC ASC device possesses better electrochemical properties than the VOOH//VOOH SSC device.

## Data accessibility

We have conducted our experiment systematically and reported the experimental procedure clearly in the experimental section, and provided all necessary data in the Results and Discussion section in the main manuscript.
